# Adipokines and Osteoarthritis: Novel Molecules Involved in the Pathogenesis and Progression of Disease

**DOI:** 10.1155/2011/203901

**Published:** 2011-08-18

**Authors:** Javier Conde, Morena Scotece, Rodolfo Gómez, Veronica Lopez, Juan Jesus Gómez-Reino, Oreste Gualillo

**Affiliations:** SERGAS, NEIRID Lab (Laboratory of NeuroEndocrine Interaction in Rheumatology and Inflammatory Diseases), Institute of Medical Research (IDIS), Santiago University Clinical Hospital Building C, Level-2, 15706 Santiago de Compostela, Spain

## Abstract

Obesity has been considered a risk factor for osteoarthritis and it is usually accepted that obesity contributes to the development and progression of osteoarthritis by increasing mechanical load of the joints. Nevertheless, recent advances in the physiology of white adipose tissue evidenced that fat cells produce a plethora of factors, called adipokines, which have a critical role in the development of ostearthritis, besides to mechanical effects. In this paper, we review the role of adipokines and highlight the cellular and molecular mechanisms at play in osteoarthritis elicited by adipokines. We also emphasize how defining the role of adipokines has broadned our understanding of the diversity of factors involved in the genesis and progression of osteoarthritis in the hope of modifying it to prevent and treat diseases.

## 1. Introduction

**Figure 1:**
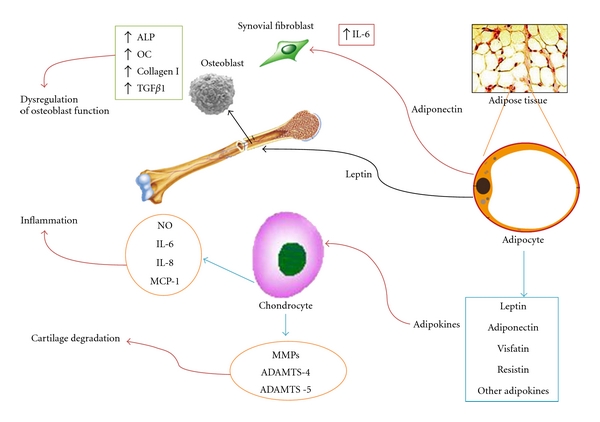
Schematic representation of the complex network that links white adipose tissue dysfunction, bone, and cartilage tissues. Dysfunctional fat produces an excess of proinflammatory adipokines that are able to interact with bone cells, synovial cells, and chondrocytes by inducing proinflammatory mediators (cytokines, ROS, NO) and cartilage degradative factors (metalloproteases and ADAMTSs).

Osteoarthritis (OA) is a multifactorial joint degenerative disease characterized by deep alteration of articular cartilage, changes in subchondral bone, osteophyte formation, and synovial inflammation. It is the most common arthritis, but its etiology is largely unknown. Age, obesity, sex, and previous injury are considered as significant risk factors. Although OA is commonly described as noninflammatory disease, inflammation is recognized as contributing to the symptoms and progression of OA [1, 2]. Inflammation may be either a primary event in OA or secondary to other aspects of disease such as biochemical changes within the cartilage. Obesity is also considered as a risk “of weight” in the pathogenesis of OA, and its contribution may be due to different convergent mechanisms that lead, as final result, to cartilage destruction, being a mechanical stress surely a risk factor for weight bearing joints [3, 4]. However, several studies demonstrated that obesity is also a risk factor for nonweight-bearing joints [5]. Obesity is nowadays considered as a chronic low-grade inflammatory status which is closely related to the release, by white adipose tissue, of a plethora of factors, most of them of proinflammatory nature, including classical cytokines such as IL-6, IL-1 and TNF-*α*, as well as adipokines, such as leptin, adiponectin, resistin, visfatin, and other recently identified factors such as chemerin, lipocalin, or serum Amiloyd 3 (SAA3) [6–8]. Adipokines are a growing family of WAT-produced factor that exert pleiotropic functions through several pathways and are involved in a wide spectrum of activities including not only glucose and lipid metabolism but also modulation of immune and inflammatory response [9]. So, certain adipokines, can be viewed as potential factors that hub obesity, inflammation, and arthritis (Figure 1). This paper will address the most relevant findings regarding the involvement of several adipokines, namely, leptin, adiponectin, resistin, visfatin, and others, in osteoarthritis.

## 2. Leptin

Leptin is a 16 kDa nonglcosylated hormone that is encoded by the obese (ob) gene, the murine homolog of human LEP gene [10]. Leptin exerts its biological actions through the activation of its OB-Rb long-form receptor isoform which is encoded by the gene diabetes (*db*) and belongs to the class 1 cytokine receptor superfamily. It is mainly produced by adipocytes, and its circulating levels are correlated with white adipose tissue (WAT) mass. Mutation in either *ob* gene, or the gene encoding the leptin receptor (the diabetes, or *db* gene), results in severe obesity. This hormone decreases food intake and increases energy consumption by acting on specific hypothalamic nuclei, inducing anorexigenic factors as cocaine amphetamine related transcript (CART) and suppressing orexigenic neuropeptides such as neuropeptide Y [11]. Leptin levels are mostly dependent on the amount of body fat, but its synthesis is also regulated by inflammatory mediators [12].

It is increasingly evident that this hormone plays a key role in the OA pathophysiology. Some initial findings have suggested an anabolic role of this hormone in the cartilage. Leptin expression is much higher in osteoarthritic human cartilage than in normal cartilage. The intra-articular injection of leptin can strongly stimulate the synthesis of insulin-like growth factor-1 (IGF-1) and transforming growth factor-*β* (TGF-*β*) at both the messenger RNA (mRNA) and protein levels which can exert anabolic activities in cartilage metabolism [13]. Leptin concentrations in synovial fluid were also significantly correlated with BMI in people with severe osteoarthritis [13]. These findings have suggested that high circulating leptin levels in obese individuals may protect cartilage from osteoarthritic degeneration. 

Nevertheless, Otero et al. have demonstrated that, in cultured human and murine chondrocytes, type 2 nitric oxide synthase (NOS2) is synergistically activated by the combination of leptin plus interferon-*γ*, and NOS2 activation by IL-1*β* is increased by leptin via a mechanism involving JAK2, PI3K, and mitogen activated kinases (MEK1 and p38) [14, 15]. Nitric oxide (NO), which is induced by a wide range of proinflammatory cytokines, is a well-known proinflammatory mediator on joint cartilage, where it triggers chondrocyte phenotype loss, apoptosis, and metalloproteinases (MMPs) activation.

Recently, it has been demonstrated that leptin is able to induce also the expression of MMPs involved in OA cartilage damage, such as MMP-9 and MMP-13 [16]. Furthermore evidences suggested that leptin alone and in combination with IL-1*β* upregulates MMP-1 and MMP-3 production in human OA cartilage through the transcription factor NF-*κ*B, protein kinase C, and MAP kinase pathways. This adipokine is also correlated positively to MMP-1 and MMP-3 in synovial fluid (SF) from OA patients [17]. Noteworthy, very recently, leptin has been demonstrated to increase IL-8 production in human chondrocytes [18]. Bao et al. have defined that leptin enhanced both gene and protein levels of catabolic factors such as MMP-2 y and MMP-9, while downregulated the anabolic factors such as basic FGF in articular cartilage of rats. Additionally, the gene expression of ADAMTS-4 and -5 were markedly increased and a depletion of proteoglycan in articular cartilage was observed after treatment with leptin. [19].

Leptin also could contribute to abnormal osteoblast function in OA. In fact, the elevated production of leptin in OA abnormal subchondral osteoblast is correlated with the increased levels of ALP (alkaline phosphatase), OC (osteocalcin), collagen type I, and TGF-*β*1 (transforming growth factor *β*1) inducing a dysregulation of osteoblast function [20]

Leptin and leptin's receptor (Ob-Rb) expression levels were significantly increased in advanced OA cartilage and in SF. The induction by leptin of IL-1*β* production, MMP-9, and MMP-13 protein expression in chondrocytes indicate a proinflammatory and catabolic role of this hormone on cartilage metabolism [21].

Ku et al. have demonstrated a relation of SF leptin concentrations with the radiographic severity of OA in OA patients, suggesting a role of leptin as an effective marker for OA [22]. 

These results suggested that leptin may act as a proinflammatory factor on cartilage metabolism, suggesting a prominent catabolic effect in OA joints. In recent studies, comparing the incidence of knee osteoarthritis between *ob*/*ob* and *db*/*db* mice and controls, no significant differences have been detected [23].

This recent finding suggested that obesity, per se, is not a sufficient condition to induce knee OA, but that leptin is necessary in the pathophysiology of OA development and progression associated with obesity. 

In fact, most studies support the role of the adipokines as a nonmechanical link between obesity and OA. In patients with clinical knee osteoarthritis, Berry et al. have demonstrated that leptin was significantly associated with increased levels of the bone formation biomarkers, such as osteocalcin and PINP, and reduced cartilage volume loss. On the contrary, baseline expression of leptin receptors OB-Rb was associated with reduced levels of the cartilage formation biomarkers PIIANP and osteocalcin, with increased cartilage defects score, and with increased cartilage volume loss [24]. All these results were independent of age, sex, and body mass index.

However, in another recent published paper, no association between leptin levels and hand OA progression has been demonstrated [25].

## 3. Adiponectin

Adiponectin, also known as GBP28, apM1, Acrp30, or AdipoQ, is a 244-residue protein that is produced mainly by WAT. Adiponectin has structural homology with collagens VIII and X and complement factor C1q, and it circulates in the blood in relatively large amounts in different molecular forms [26, 27].

It increases fatty acid oxidation and reduces the synthesis of glucose in the liver. Ablation of the adiponectin gene has no dramatic effect on knockout mice on a normal diet, but when placed on a high fat/sucrose diet, they develop severe insulin resistance and exhibit lipid accumulation in muscles. Circulating adiponectin levels tend to be low in morbidly obese patients and increase with weight loss [26, 27].

Adiponectin acts via two receptors, one (AdipoR1) found predominantly in skeletal muscle and the other (AdipoR2) in liver. Transduction of the adiponectin signal by AdipoR1 and AdipoR2 involves the activation of AMPK, PPAR-*α*, PPAR-*γ*, and other signalling molecules [27].

Actually, some evidences indicate that adiponectin has a wide range of effects in pathologies with inflammatory component, such as cardiovascular disease, endothelial dysfunction, type 2 diabetes, metabolic syndrome, and OA [28]. In contrast to its previously described protective role in vascular diseases, there are some evidences that adiponectin might act as a proinflammatory factor in joints, and it could be involved in matrix degradation. 

Adiponectin-treated chondrocytes lead to the induction of NOS2, via a signalling pathway that involves PI3 kinase. Similarly, this adipokine also increases IL-6, MMP-3, MMP-9, and MCP-1 production in the same cell type [29]. Very recently, the induction of MMP-3 by adiponectin in chondrocytes was further confirmed, and it occurred, in part, through p38, AMPK, and NF-*κ*B [30]. In addition, Kang et al. have reported that collagenase-cleaved type II collagen neoepitope, a product of collagen type II degradation, was increased in supernatants of adiponectin-induced OA cartilage explants [31]. Furthermore, it has been reported that adiponectin is able to induce the expression of IL-6 in human synovial fibroblasts [32]. 

Very recently, Frommer and colleagues confirmed the proinflammatory role of adiponectin in RA. These authors demonstrated that adiponectin induces gene expression and protein synthesis of chemokines and proinflammatory cytokines in RA effector cells, such as synovial fibroblasts, lymphocytes, chondrocytes, and endothelial cells. In fact, adiponectin promoted inflammation through cytokine synthesis, attraction of inflammatory cells to the synovium, and recruitment of prodestructive cells via chemokines, thus promoting matrix destruction at sites of cartilage invasion [33].

Filkova et al. found higher adiponectin serum levels in erosive OA patients compared with nonerosive OA patients [34]. In the same way, Distel and colleagues have shown that there was an increase in IL-6 and adiponectin production in infrapatellar fat pad in knee osteoarthritis [35]. Taken together, these results suggest that adiponectin may be considered a potential molecule involved in joint disorders and matrix degradation.

However, the role of adiponectin in OA is controversial. There are certain evidences that show an inhibition of IL-1*β*-induced MMP-13 expression and upregulation of tissue inhibitor of metallopreoteinase-2 (TIMP-2) mediated by adiponectin in chondrocytes [36]. Moreover, in STR/Ort mice, an animal osteoarthritis model, the serum adiponectin levels are lower compared with control group [37], suggesting a protective role for this adipokine in the development of the disease.

To note, clinical data also support the fact that adiponectin could be a protective molecule against OA. A very recent study developed by Honsawek and Chayanupatkul showed an inverse correlation between adiponectin and disease severity [38]. Moreover, it has been reported that patients with high adiponectin levels had a decreased risk for hand OA progression, suggesting that this adipokine may be a protective hormone against cartilage damage [25].

## 4. Visfatin

Visfatin, also called PBEF (pre-B-cell colony-enhancing factor) and Nampt (nicotinamide phosphoribosyltransferase), is a protein of approximately 471 amino acids and 52 kDa [39]. It is a hormone that originally was discovered in liver, bone marrow, and muscle, but it is also secreted by visceral fat [39, 40].

It has been reported that visfatin is increased in obesity. Moreover, leucocytes from obese patients produce higher amounts of visfatin compared with lean subjects, and specifically, granulocytes and monocytes are the major producing cells [41, 42]. Macrophages have been described as a source for visfatin production too [43].

It is supposed that visfatin has insulin mimetic properties, however the role of this adipokine in glucose metabolism is still unclear [40, 44]. Visfatin is upregulated in models of acute injury and sepsis [45], and its synthesis is regulated by other factors such as glucocorticoids, TNF-*α*, IL-6, and growth hormone (GH). Moreover, visfatin has been shown to induce chemotaxis and the production of IL-1*β*, TNF-*α*, and IL-6 in lymphocytes [46]. 

Recently, Gosset and colleagues have reported that IL-1*β* was able to increase visfatin expression in human cultured chondrocytes [47]. Similarly, visfatin-treated chondrocytes showed an increase in MMP-3, MMP-13, ADAMTS-4, and ADAMTS-5 expression, while aggrecan production was reduced [47], suggesting a prodegradative and catabolic role of visfatin in articular cartilage.

Another recent study shows that infrapatellar fat pad from OA patients release high amounts of visfatin [48] and based on the previous data which suggest that visfatin is able to induce matrix metalloproteinases, authors concluded that visfatin secreted by infrapatellar fat pad might contribute to the pathophysiological changes occurred in OA.

Moreover, Duan and colleagues have reported that OA patients have increased levels of visfatin in synovial fluid, and this adipokine is positively correlated with degradation biomarkers of collagen type II and aggrecan, demonstrating that visfatin is involved in matrix degradation [49].

## 5. Resistin

Resistin is a macrophage/monocyte-derived proinflammatory mediator [50]. It belongs to the found in inflammatory zones (FIZZ) family (also known as RELMs, resistin-like molecules). It was secreted by adipose tissue, but it has been found also in macrophages, neutrophils, and other cell types. Serum resistin levels increase with obesity in mice, rats, and humans [51, 52]. The proinflammatory profile of resistin, together with its association with obesity suggest that this adipokine might be another potential mediator that links OA with inflammation and obesity. It was demonstrated that this adipokine is elevated in both serum and SF after traumatic joint injuries. Recombinant resistin stimulated proteoglycan degradation in mouse femoral head cultures, the induction of inflammatory cytokines, and PGE2 production. Moreover, it inhibited proteoglycan synthesis in human cartilage explants [53]. However, Gómez et al. have not identified an association between baseline serum levels of resistin and cartilage volume loss [18].

## 6. Other Adipokines

*Chemerin,* also known as tazarotene-induced gene 2 and retinoic acid receptor responder 2 (RARRES2), is a novel chemoattractant adipokine [54]. It is secreted as an 18 kDa inactive proprotein and activated by posttranslational C-terminal cleavage [55]. Chemerin acts via the G-coupled receptor chemokine-like receptor 1 (CMKLR1) [54]. Chemerin and its receptor are mainly expressed, but not exclusively, in adipose tissue [56]. For instance, dendritic cells and macrophages express chemerin receptor [57]. Moreover, chondrocytes express chemerin and its receptor [8, 58] and IL-1*β*, along with other adipokines and glucocorticoids, is able to modulate the expression of this adipokine [8], demonstrating the importance of chemerin in chondrocyte pathophysiology, being this molecule regulated by others, which drive inflammatory processes as IL-1*β*, leptin, LPS, and so forth.

*Lipocalin 2* (LCN2), also termed siderocalin, 24p3, uterocalin, and neutrophil gelatinase-associated lipocalin, is a 25 kDa glycoprotein isolated from neutrophil granules, although adipose tissue is thought to be the main source [59]. The LCN2 protein has been isolated as a 25 kDa monomer, as a 46 kDa homodimer and in a covalent complex with MMP-9, and its cellular receptor, megalin (GP330), was recently described [60]. LCN2 is involved in apoptosis of haematopoietic cells [61], transport of fatty acids and iron [62], and modulation of inflammation [63] among other processes. 

LCN2 has recently been identified in chondrocytes [64]. In these cells IL-1*β*, leptin, adiponectin, LPS, and dexamethasone act as potent modulators of its expression [8]. LCN2 is likely to be involved in matrix degradation since it forms molecular complexes with MMP-9 [65].

*Serum Amyloid A3* (SAA3) protein is an adipokine that belongs to the family of acute-phase serum amyloid A proteins (A-SAA) secreted in the acute phase of inflammation. In mice, all A-SAA proteins are actively transcribed [66–68], whereas in humans, SAA3 is encoded by a pseudogene and its functional protein is unknown [69, 70].

Mouse SAA3 is involved in metabolic, immune, and cardiovascular homeostasis [71–73]. It has been reported that SAA3 is induced in rabbit primary chondrocytes and it can induce MMP-13 transcription [74]. Furthermore, very recently SAA3 expression was described in murine chondrocytes, where it was regulated by cytokines such as IL-1*β*, leptin, or adiponectin [8].

*Vaspin* is a visceral adipose tissue-derived serpin with potential antiprotease properties [75]. The administration of this factor to obese mice improved glucose tolerance and insulin sensitivity and reversed altered expression of genes that might promote insulin resistance. 

*Omentin* is a protein of 40 kDa, secreted by omental adipose tissue and it is highly abundant in human plasma. Omentin has previously been identified as intelectin, a new type of Ca2+-dependent lectin. Several studies have shown that omentin gene expression is alternated by inflammatory states and obesity [76].

Recently, Šenolt et al. have demonstrated increased levels of vaspin and reduced levels of omentin in the synovial fluid of patients with RA compared with those with OA [77]. This finding suggests that these two adipokines are likely involved in OA pathophysiology.

## 7. Conclusions

It is now clear that adipokines have multiple relevant roles in the body, and many research efforts are driven to elucidate the intricate network among WAT, metabolic disorders, and inflammatory diseases. Although, many aspects are still unclear, in this paper we summarize the present knowledge on the role of adipokines in OA pathophysiology.

However, the present knowledge is almost incomplete to allow translation of these approaches to clinical practice, several potential approaches are likely feasible. For instance, the control of leptin levels by using antibodies in a similar way to anti-TNF therapy might be an interesting strategy.

Nevertheless, apart from pharmacological perspectives, it is clear that the main source of all proinflammatory adipokines is the dysfunctional adipose tissue. Therefore, reducing fat mass, overeating, and increasing physical activity remain the essential preventive strategy to counteract the negative effects of obesity proinflammatory state.

Only further insights that clarify the mechanisms by adipokines are regulated and which are the concrete roles of them in the OA pathology, could propose new pharmacological approaches for this disease.
